# Supplementary material for: Higher Availability of Long-Chain Monounsaturated Fatty Acids in Preterm than in Full-Term Human Milk
Source: Life (Basel). 2023 May 17;13(5):1205. doi: 10.3390/life13051205 (PMC10223021; doi:10.3390/life13051205)
Supplement: Supplementary file 1 [file life-13-01205-s001.zip › life-2279195-supplementary.pdf]

Higher availability of long-chain monounsaturated fatty acids in preterm than in full-term human milk

Tamás Marosvölgyi[1,2*], Timea Dergez[1], József L. Szentpéteri[3], Éva Szabó[2,4*] and Tamás Decsi[2]

[1] Institute of Bioanalysis, Medical School, University of Pécs, Pécs, Hungary
[2] Department of Paediatrics, Medical School, University of Pécs, Pécs, Hungary

[3] Institute of Transdisciplinary Discoveries, Medical School, University of Pécs, Pécs, Hungary

[4] Department of Biochemistry and Medical Chemistry, Medical School, University of Pécs, Pécs, Hungary

Table S1: Preterm (PT) and full-term (FT) human milk weighted least squares mean contents of individual long-chain monounsaturated fatty acids (LCMUFA) and estimated LCMUFA across lactation stages in weight%(g/100 g) based on Table 2 and 3 in [29]

Lactation stage		C20:1n-9		C22:1n-9		C24:1n-9		Estimated LCMUFA
Mean ± SEM		k (N)	weight %	k (N)	weight %	k (N)	weight %	weight %
C (0 – ≤5 d)	PT (≤37 w)	4 (50)	0.66 ± 0.02	5 (282)	0.16 ± 0.00	7 (328)	0.29 ± 0.01	1.11
	FT (≥37 – ≤42 w)	13 (470)	0.88 ± 0.07	9 (374)	0.22 ± 0.02	12 (502)	0.28 ± 0.04	1.38
TM (6 – ≤15)	PT (≤37 w)	4 (70)	0.50 ± 0.01	6 (360)	0.12 ± 0.02	6 (318)	0.14 ± 0.00	0.76
	FT (≥37 – ≤42 w)	12 (553)	0.60 ± 0.05	11 (513)	0.21 ± 0.07	9 (415)	0.27 ± 0.12	1.08
MHM (16 – ≤60 d)	PT (≤37 w)	2 (26)	0.44 ± 0.06	5 (298)	0.08 ± 0.00	5 (305)	0.04 ± 0.00	0.56
	FT (≥37 – ≤42 w)	24 (1768)	0.45 ± 0.03	20 (1697)	0.11 ± 0.01	20 (1532)	0.07 ± 0.01	0.63

Fatty acid values are given in the mean ± SEM form.

Abbreviations: C: colostrum, k: number of included studies, MHM: mature milk, N: number of participating mothers, TM: transitional milk
